# Case of arthritis secondary to leprosy

**DOI:** 10.1186/2193-1801-3-734

**Published:** 2014-12-15

**Authors:** Fiaz Alam, Samar AL Emadi

**Affiliations:** Hamad Medical Corporation, P.O. Box 3050, Doha, Qatar; Hamad Medical Corporation, WCMC-Q, P.O. Box 3050, Doha, Qatar; Affiliated with Weill Cornell Medical College, Doha, Qatar

## Abstract

**Introduction:**

Leprosy is a chronic granulomatous infectious disease, which is caused by *Mycobacterium leprae*. High numbers of people are still affected by this disease in some of the developing countries however, it is rarely seen in non-endemic regions.

Cutaneous and neurological manifestations are the common and classical presentations of leprosy. Musculoskeletal involvement is the third most common manifestation but is less frequently reported.

Joint involvement can present as acute symmetrical polyarthritis or chronic polyarthritis resembling rheumatoid arthritis. Leprosy can also present with tenosynovitis, either isolated or associated with arthritis.

**Case presentation:**

We report a case of 29-year-old man who developed tenosynovitis and acute symmetrical polyarthritis including small joints of hands and feet three weeks after appearance of typical cutaneous lesion of leprosy. Patient improved with steroid and anti leprosy treatment. Patient had another acute episode with symmetrical polyarthritis four months later while on treatment.

**Discussion and Evaluation:**

In the modern era, there is increase movement of population from developing countries to developed countries, it is likely that patient suffering from leprosy with arthritis may present to rheumatology clinic in those countries where leprosy is not endemic. Exact pathogenesis for musculoskeletal manifestations is still not fully known. Our patient presented with symmetrical polyarthritis, enthesitis and systemic involvement, which was secondary to lepra reaction.

Having good knowledge of musculoskeletal manifestation of leprosy, will help narrow differential diagnosis and will prevent unnecessary diagnostic workup.

**Conclusion:**

Leprosy can present with different rheumatologic manifestation including tenosynovitis and acute symmetrical polyarthritis. Type 2 lepra reaction (Erythema nodosum leprosum ENL) can present with systemic manifestation and can involve skin, nerves, joints, kidneys and liver.

## Introduction

Leprosy is an infectious disease caused by Mycobacterium leprae. It is an important global health concern and needs early diagnosis and prompt treatment to prevent permanent disability.

Leprosy is multisystem granulomatous disease. It primarily involves the skin and peripheral nerves. The severity of neurological and cutaneous presentation depends upon the number of bacilli and type of immune response. It could be either pauci bacillary with intense immune reactions or multibacillary with weak immune response.

### Epidemiology

115 countries submitted their reports to WHO (World health organization 2014) by the end of 2012. The global registered prevalence of leprosy at the end of 2012 was 189 018 cases. The number of new cases reported globally in 2012 was 232 857 compared with 226 626 in 2011.

The global statistics (World health organization) show that 220 810 (95%) of new leprosy cases were reported from 16 countries and only 5% of new cases are from the rest of the world.

There are two different ways of classifying leprosy.

Ridley-Jopling (Pardillo et al. 2007) system and the simpler and more commonly used WHO classification system.

### Ridley-Jopling classification

**Figure 1:**
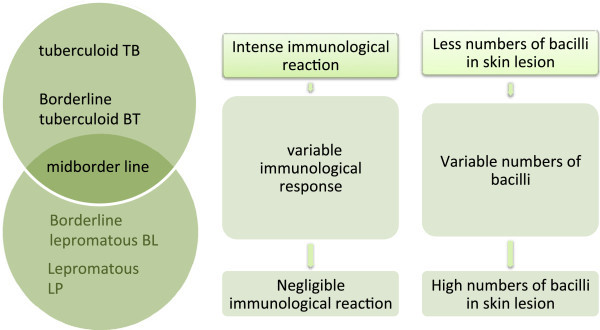
**Ridley-Jopling classification.**

Leprosy is classified (Pardillo et al. 2007) into five different forms depending upon the immunological reaction and number of bacilli in skin lesions. Less number of bacilli in skin lesions and severe immunological reaction characterizes tuberculoid leprosy while lepromatous leprosy exhibits weak immunologic reaction and increase number of bacilli in skin lesions. These five different forms are Polar tuberculoid form, Borderline tuberculoid, Mid-borderline, Borderline lepromatous, and Polar lepromatous leprosy (Figure 1).

### WHO classification

The WHO (Pardillo et al. 2007; WHO 2011) classifies leprosy according to the number of lesions and the presence of bacilli on a skin smear.

Paucibacillary (PB) leprosy is defined as five or fewer skin lesions. Bacilli are not seen on skin smears. Patients with only a single skin lesion are classified separately as single lesion PB leprosy. Multibacillary (MB) leprosy is defined as six or more lesions. In case of MB skin smear may be positive for bacilli.

If only number of skin lesion are considered then it can lead to overtreatment of paucibacillary leprosy as skin smear may be negative in patients with more then 5 skin lesion.

Musculoskeletal (Shejpal et al. 2014; Wakhlu and Agarwal 2010; Prasad et al. 2012; Atkin et al. 1990, 1987; Lopéz et al. 2012) manifestations are common in leprosy but less frequently reported. Joint involvement occurs in about 75% of cases of leprosy.

Different patterns of musculoskeletal presentation can occur in leprosy patient. Joint involvement can occur in form of monoarticular, oligoarticular or polyarticular. It can present as acute (Atkin et al. 1987) symmetrical polyarthritis or chronic symmetrical polyarthritis mimicking rheumatoid arthritis (Lopéz et al. 2012). Enthesitis (Atkin et al. 1990) and tenosynovitis are also seen in leprosy patient.

## Case presentation

27 year old Nepali male presented to the emergency department with five days history of high grade fever, multiple joint pain and swelling preceded by four weeks history of progressive painful nodular skin lesion over the face, scalp, both ears, all four limbs and upper chest. Fever was not associated with headache, productive cough, abdominal pain, dysuria and diarrhea. Small joints of hands, bilateral elbows, knees and feet were painful. There was no history of preceding diarrhea, upper respiratory tract infection or urinary tract infection.

**Figure 2:**
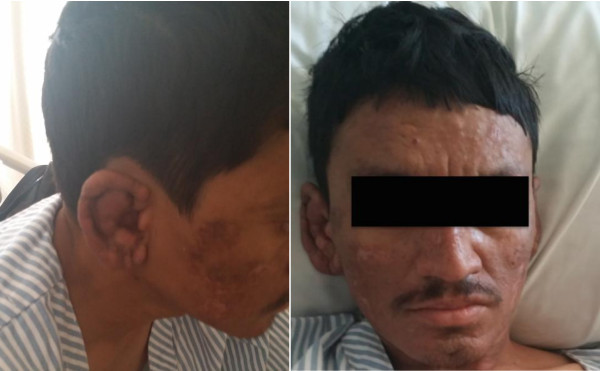
**Papulo-nodular skin lesions over the face and ear.**

There were no previous similar complaints in the past.On examination patient was febrile with temperature of 38 centigrade, blood pressure of 114/74, pulse 95 per minute. His general appearance revealed multiple painful nodular skin lesions over face, both ears, upper and lower limbs. (See Figure 2). There were widespread hypo and hyper-pigmented skin lesions. Sensation over the skin lesion was intact.

Musculoskeletal examination revealed tender and swollen bilateral MCPS, PIPs, wrists, elbows, bilateral knees and bilateral 3^rd^ and 4^th^ MTPs. Patient also had evidence of enthesitis around both knees at site of quadriceps tendon insertion and tenosynovitis of wrist extensors bilaterally. Lung and cardiovascular examination was essentially normal. Neurological examination showed thickened ulnar nerves bilaterally with no evidence of ulnar neuropathy.

Cranial nerves were intact. Muscle power in all groups of muscles was normal. There was no sensory impairment at any level.

Laboratory investigations revealed WBC count of 36800 (normal 4–10). (93% neutrophils), Hemoglobin (Hb) of 8.7 g/dl (MCV of 74.6, MCH of 22.3), Platelet counts of 656 K; blood urea nitrogen 13.6 mmol/L (normal 2.5 -6.7) and creatinine 163umol/L normal (50.4-98.1). Inflammatory markers were raised (CRP of 335 mg/l and ESR of 87 mm/1 hr). Urine chemical examination showed 2+ protein and 3+ erythrocytes.

Urine Microscopy revealed WBC count of 103 and Red blood cell of 486 with positive granular cast.

Urinary protein creatinine ratio was 117 mg/mmol (normal range of 0–45).

Blood and urine culture didn’t grow any organism.

**Figure 3:**
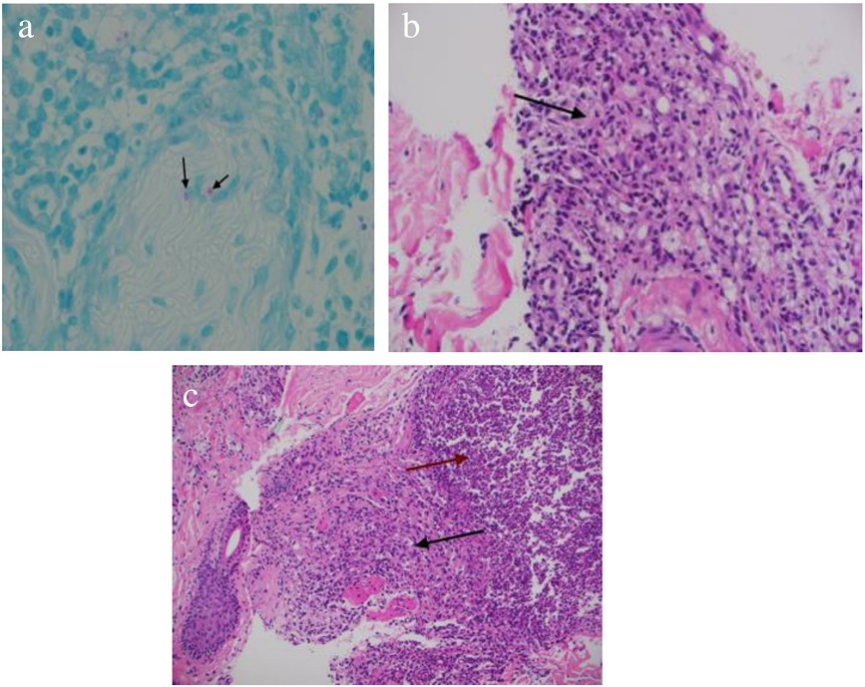
**Histopathology of skin nodule.** **(a)** Oil immersion wade fite stain showing acid-fast bacilli (arrows), **(b)** H&E stain x 400 magnification showing granuloma (black arrow), **(c)** H&E stain x 200 magnification showing granuloma (black arrow) and acute folliculitis with neutrophillic infiltrates (red arrow).

Further investigation revealed negative ANA, RF, anti ccp and normal complements. X rays of hands and feet were normal.Punch skin biopsy (see Figure 3 histopathology of skin nodule) was taken from the nodule present over right upper arm. Skin biopsy revealed non-necrotizing granuloma with numerous foamy macrophages in dermis and subcutaneous fat. Small number of acid-fast bacilli was seen in some of the macrophages and biopsy result was reported as borderline tuberculoid. Leprosy was diagnosed on basis of typical skin lesion and skin biopsy finding.

Treatment of our case included intravenous hydration and anti-leprosy medication (rifampicin 600 mg monthly and dapsone 100 mg daily). Skin lesions and arthritis initially got worse after starting treatment, which improved with addition of high dose steroid, (Prednisolone 60 mg daily). Polyarthritis resolved five days after starting treatment with multidrug anti-leprosy medication and high dose steroid. Patient renal function returned to normal. Patient didn’t undergo renal biopsy after improvement in his renal function. Patient was discharged in good general condition.

Patient had regular follow up in the out patient clinic with infectious disease doctor. Steroid was tapered slowly and patient was improving clinically. Four months after initiation of treatment, patient suddenly developed similar acute presentation. Patient was receiving monthly rifampicin 600 mg and daily dapsone of 100 mg. Patient landed in emergency department with sudden onset of high grade fever, new skin painful nodular lesions, symmetrical polyarthritis involving small joints of hands, bilateral elbow joint, bilateral knees, ankles and bilateral metatarsophalangeal joints.

Laboratory investigations revealed leukocytosis, high inflammatory markers, and raised liver enzymes.

This episode was considered to be secondary to type 2-lepra reaction and treated accordingly. Patient responded well to 20 mg of daily prednisolone. Patient is regularly followed up in infectious disease outpatient clinic and is receiving combined antileprosy medication for paucibacillary leprosy.

## Discussion

Leprosy is a disease, which is more common in endemics areas and developing countries. Because of increase travel in today’s world, it is possible that we may come across with leprosy patient in non-endemic areas. It is important to be updated about different manifestation of leprosy in order to avoid unnecessary diagnostic tests and to start proper and early treatment.

Exact pathogenesis of joint involvement in leprosy is still not fully elucidated. Lepra reactions (Graham et al. 2010; Bhat and Prakash 2012; Wakhlu and Agarwal 2010) (Types I and II lepra reaction), and direct infiltration (Wakhlu and Agarwal 2010) of the synovium by mycobacterium leprea are thought to be the underlying pathogenesis mechanisms for joint involvement. Peripheral sensory neuropathy (Wakhlu and Agarwal 2010) can also lead to Charcot’s neuropathy.

There are two types of immunologic reactions to mycobacterium leprae antigens. These immunologic reactions can occur before, during or after the treatment of leprosy.

Type 1 Lepra (Graham et al. 2010; Bhat and Prakash 2012) reaction is a delayed hypersensitivity reaction. It is cell mediated immune response to *M. leprae* antigenic determinants and is characterized by acute inflammation of pre-existing skin lesions or by the appearance of new lesions and/or neuritis. Systemic involvement does not occur in type 1 reation.

Type 2 lepra (Graham et al. 2010; Bhat and Prakash 2012) reaction also termed as Erythema nodosum leprosum (ENL) is an immune complex mediated (type 3 hypersensitivity) response to M.leprae antigenic determinants. It results in severe painful skin lesion, nerve damage, fever and systemic manifestation. Systemic involvement can lead to arthritis, dactyltis, orchtitis, uveitis, lymphadenitis, glomerulonephritis, protienuria and hepatitis. It occurs mostly during the fist year of leprosy treatment. Recurrence of type 2 reaction is also common during treatment.

Chauhan et al (Wakhlu and Agarwal 2010) classify the arthritis in leprosy into the following groups: (1) Charcot’s arthropathy secondary to peripheral sensory neuropathy; (2) swollen hands and feet syndrome; (3) acute polyarthritis of lepra reaction; and (4) chronic arthritis from direct infiltration of the synovium by lepra bacilli.

Shiva PRASAD (Prasad et al. 2012) and his team conducted retrospective case series study of leprosy patients. Forty-four cases with leprosy were identified. Musculoskeletal manifestations included arthritis (n = 22), swollen hands and feet syndrome (n = 11), tenosynovitis (n = 9), painful swollen feet (n = 9), arthralgia (n = 7) and vasculitis (n = 1). Arthritis and tenosynovitis were part of spontaneous onset lepra reaction in 28 cases.

S L Atkin et al. (Atkin et al. 1990) conducted a study in seventy-seven patients with leprosy. Study showed that ten patients had generalized enthesitis and twenty patients had leprosy with manifestations of arthritis.

Another study, conducted by S L Atkin (Atkin et al. 1987) in patients with leprosy in Papua New Guinea, showed that 31 patients out of 55 had peripheral symmetrical inflammatory arthritis.

Hence it is important to include leprosy in list of possible differential diagnosis of arthritis, mainly in those countries where leprosy is prevalent or those patients who travelled from endemic areas.

Our patient’s presentation with peculiar skin lesion combined with acute polyarthritis leads to diagnosis of leprosy.

Our patient presented with an acute onset of symmetrical polyarthritis and high-grade fever. High inflammatory markers, leukocytosis (Legendre et al. 2012) negative blood and urine cultures are supporting evidence for type 2 lepra reaction. Acute arthritis as well as enthesitis and tenosynovitis improved within a week after initiation of treatment.

Sometime, it is difficult to differentiate symmetrical polyarthritis due to leprosy from rheumatoid arthritis as in our patient. This question was entertained upon initial presentation of our patient with symmetrical polyarthritis. Absence of rheumatoid nodule, extra articular manifestation, patient being male, good response to anti leprosy treatment, and absence of Rheumatoid factor and anti-ccp are the clinical distinguishing features from rheumatoid arthritis in our patient.

At presentation patient also had acute renal impairment, which improved with hydration. Wide variety of renal involvement (Ahsan et al. 1995; Aggarwal et al. 2004) occurs in leprosy. Glomerular and interstitial lesions both can happen in leprosy. Glomerulonephritis, acute interstitial nephritis, tubulointerstitial nephritis, acute tubular necrosis and amyloidosis are important renal manifestation of leprosy.

Clinical feature of glomerulonephritis due to leprosy consist of asymptomatic hematuria and proteinuria. In some cases patient can also present with nephritic clinical picture. Immune complex mediated glomerulonephritis is the most common form of glomerulonephritis. ENL type 2 lepra (Ahsan et al. 1995; Aggarwal et al. 2004; Singhal et al. 1977) reaction is immune complex type 3 hypersensitivity reaction which can lead to immune complex mediated glomerulonephritis in leprosy.

Our patient had mild acute renal impairment with transient hematuria and proteinuria. In the presence of systemic manifestation, transient renal impairment was most likely secondary to type 2 lepra reaction.

The other interesting part in our case was second presentation, which was acute in onset. In spite on multidrug treatment and low dose steroid, patient developed this acute presentation. Patient developed new skin lesions, fever and polyarthritis. Laboratory investigation showed leukocytosis and raised liver enzyme. Question was raised whether patient has recurrence of leprosy disease or simply a lepra reaction. Possibility of drug induced liver injury was also entertained. It is interesting to note that recurrence (Graham et al. 2010; Bhat and Prakash 2012) of type 2 lepra can happen while patient on treatment. Type 2-lepra reaction can present with all these systemic manifestation including raised liver enzymes. Patient was managed as having type 2 lepra reaction and responded well.

## Conclusion

Musculoskeletal manifestations are common in leprosy.

Leprosy should be included in differential diagnosis in those patients who present with typical skin rash and musculoskeletal manifestation. Type 2 lepra reaction, ENL, can be recurrent and usually presents with systemic manifestation involving skin, nerves, joint, kidney and liver as it happened in our patient.

Diagnosis of leprosy is easy but need to be thought off especially in migratory population to non-endemic areas.
